# Insulin Receptor Substrate Gene Knockdown Accelerates Behavioural Maturation and Shortens Lifespan in Honeybee Workers

**DOI:** 10.3390/insects10110390

**Published:** 2019-11-05

**Authors:** Kate E. Ihle, Navdeep S. Mutti, Osman Kaftanoglu, Gro V. Amdam

**Affiliations:** 1 Honey Bee Breeding, Genetics, and Physiology Laboratory, USDA-ARS Baton Rouge, LA 70820, USA; 2 School of Life Sciences, Arizona State University, Tempe, AZ 85287, USAOsman.Kaftanoglu@asu.edu (O.K.); Gro.Amdam@asu.edu (G.V.A.); 3 Department of Ecology and Natural Resource Management, Norwegian University of Life Sciences, 1430 Aas, Norway

**Keywords:** insulin signalling, foraging, *Apis mellifera*, lifespan regulation, behavioural maturation

## Abstract

In animals, dietary restriction or suppression of genes involved in nutrient sensing tends to increase lifespan. In contrast, food restriction in honeybees (*Apis mellifera*) shortens lifespan by accelerating a behavioural maturation program that culminates in leaving the nest as a forager. Foraging is metabolically demanding and risky, and foragers experience increased rates of aging and mortality. Food-deprived worker bees forage at younger ages and are expected to live shorter lives. We tested whether suppression of a molecular nutrient sensing pathway is sufficient to accelerate the behavioural transition to foraging and shorten worker life. To achieve this, we reduced expression of the *insulin receptor substrate* (*irs*) gene via RNA interference in two selected lines of honeybees used to control for behavioural and genetic variation. *irs* encodes a membrane-associated protein in the insulin/insulin-like signalling (IIS) pathway that is central to nutrient sensing in animals. We measured foraging onset and lifespan and found that suppression of *irs* reduced worker bee lifespan in both genotypes, and that this effect was largely driven by an earlier onset of foraging behaviour in a genotype-conditional manner. Our results provide the first direct evidence that an IIS pathway gene influences behavioural maturation and lifespan in honeybees and highlight the importance of considering social environments and behaviours when investigating the regulation of aging and lifespan in social animals.

## 1. Introduction

Dietary restriction, a reduction in nutrient intake without malnutrition, can increase lifespan in many animals [1,2,3]. Reduced protein intake is especially effective in lifespan extension [3,4]. Similar effects are observed with molecular and pharmacological interventions that suppress the conserved, nutrient sensing insulin/insulin-like signalling (IIS) and target of rapamycin (TOR) pathways [5,6,7]. Signalling through IIS/TOR pathways is upregulated in response to food intake and high nutrient stores [8,9], and can lead to further changes in food-related behaviours [10,11,12]. Dietary restrictions and IIS/TOR suppression may involve the same mechanisms to extend lifespan, with nutrient sensing mediating the effects of dietary restriction on aging and longevity [13,14].

These mechanisms of lifespan extension appear to be broadly conserved, but their interplay with behaviour is not well understood. In the honeybee (*Apis mellifera*), the IIS pathway is thought to be a central regulator of social behaviours including the onset of foraging and the type of food collected [11,15,16,17,18,19]. Worker honeybees have an age-associated division of labour system in which younger workers called “nurses” tend the brood in the nest and older workers forage [20]. The “foragers” leave the nest to collect floral nectar and pollen, the colony’s primary sources of dietary carbohydrates and protein respectively. Genes in the IIS pathway tend to be expressed differently in the brains and fat bodies of nurses and foragers [15,21,22,23] and suppression of the *insulin receptor substrate* (*irs*) gene, which is central to IIS, results in foragers that collect more pollen [11]. Regulation of behavioural maturation is important for understanding aging and lifespan in honeybees, as it is a strong predictor of total lifespan [24]. Foragers experience increased rates of physiological and cognitive aging [25,26], explaining why bees that forage at young ages live shorter lives than those that begin to forage later in life [24,27].

Consistent with findings in other animals, dietary restriction can extend worker lifespan in the laboratory. Several studies have found that workers caged in small groups live longest on diets high in carbohydrates and low, or even lacking, in protein [28,29,30,31]. However, dietary effects on individual worker lifespan appear to be highly dependent on experimental setup, protein source and accessibility, as several other studies have instead found that caged workers live longest when given various pollens in addition to a carbohydrate source [32,33,34]. An important consideration when interpreting these results is that caged bees are deprived of the social context of a colony and the opportunity to fully express their behavioural repertoire. In the colony, the behavioural role of workers is closely associated with nutritional status [35,36,37]. Nurses consume diets rich in amino acids and have large energy stores and circulating levels of vitellogenin (Vg), a nutrient-sensitive yolk precursor and major storage protein that inhibits foraging [38,39,40,41]. Older foragers, in contrast, subsist primarily on carbohydrates and have reduced fat and protein stores and levels of Vg in their haemolymph [36,39,42]. The connection between nutritional status and behaviour, moreover, appears to be causal: Depleting the nutrients stores of colonies or individual bees triggers early foraging behaviour [35,36,37], as does RNA interference (RNAi) mediated knockdown of *Vg* [41,43,44]. Increasing individual amino acid stores has the opposite effect [17].

Dietary restriction in colony-living worker honeybees, in other words, changes physiology and accelerates a behavioural transition that leads to aging and a shorter life. The expression of IIS genes correlates with this transition [15,22], and the *irs* gene is causally involved in foraging decisions [11]. Otherwise little has been directly demonstrated about the role of IIS in honeybee lifespan regulation. Honeybees store nutrients primarily in the fat body, a tissue analogous to vertebrate liver and white adipose tissue [45]. Consistent with the general association between IIS activity and nutrient status, the expression of the two honeybee *insulin-like peptide* (*AmIlp*) genes is elevated in the fat body of well-nourished nurse bees compared to the nutrient-depleted foragers [22,31]. Amino acid supplementation increases the expression of *AmIlp1* in the fat body [17], while the same peptide gene appears to show the opposite trend in the brain [15].

We hypothesized that suppression of the IIS pathway would induce an early onset of foraging behaviour and thereby reduce the lifespan of worker honeybees. To test this hypothesis, we used RNAi to knock down *irs* gene expression in the fat body of adult bees and measured foraging onset and lifespan. We took advantage of the experimental approach of Wang and colleagues [11] who used divergently selected lines of honeybees, the high and low pollen hoarding strains [46,47], to demonstrate an effect of *irs* on the foraging loading and bias for carbohydrate (nectar) versus protein (pollen) collection. These selected honey bee lines allowed us to reduce behavioural and genetic variation, which can permit more reliable detection of single gene effects on complex behaviour [11].

## 2. Materials and Methods

### 2.1. dsRNA Preparation

We prepared double-stranded RNA toward both *irs* and green fluorescent protein (*GFP*) as described before [11,48]. Briefly, for dsRNA toward *irs* we used a fragment of the *irs* open reading frame cloned using forward and reverse primers 5′-TTTGCAGTCGTTGCTGGTA-3′; 5′-GCTTAAAGCCGGATAACGTG-3′, respectively, into the pCR^®^ 4-TOPO^®^ vector as a template for PCR [11]. PCR primers fused to T7 promoter sequence (underlined)

F: 5′-TAATACGACTCACTATAGGGCGAGCGAACCGGTAGTCGTAAAG-3′ and R: 5′-TAATACGACTCACTATAGGGCGAGCAGTGATCAAACGTGGCTT-3′ were used to produce a 583 bp product. *GFP* dsRNA was synthesized from AF09833 as a template as described before [41,48,49]. Underlined segments specify the T7 promotor sequences fused to the *Vg*-specific primers. We purified the resulting PCR products using the QIAquick PCR purification kit (Qiagen, Valencia, CA, USA.). dsRNA was then prepared using the AmpliScribe T7 transcription kit (Epicentre Biotechnologies, Madison, WI, USA.). We purified the dsRNA with phenol: chloroform extraction and verified product size and purity on a 1% agarose gel. dsRNA was brought to a final working concentration of 10 μg/μL in nuclease-free water [11].

#### 2.2. Bees

We used worker bees from the high and low pollen hoarding strains developed by Robert Page and M. Kim Fondrk [46,47]. These bees were artificially, bidirectionally selected for levels of pollen stores in their colonies, which also reduced genetic and behavioural diversity within the strains relative to the general population. The original genetic material for the stocks came from several large commercial beekeeping operations and were periodically outcrossed to maintain genetic diversity [46,47]. Bees were maintained at the Honeybee Research Laboratory in Mesa, AZ at the Arizona State University Polytechnic Campus. Queens from three high pollen hoarding strain and three low pollen hoarding source colonies were caged overnight on a single frame of wax cells and allowed to lay eggs for 24 h. This allows for collection of same-aged newly-emerged bees. The frames were then removed from their source colonies, marked to indicate that source, and placed in wild-type colonies where they were co-fostered. We removed frames from the colonies 20 days after the queens were caged and allowed the worker bees to emerge in an incubator set at 34 °C and 80% relative humidity.

Newly emerged bees from each strain were brushed from their frames into a pool and randomly assigned to one of three treatment groups: (1) the non-injected reference group (NoI), (2) the injected-control group which received dsRNA against *GFP* (GFP), and (3) the *irs* knockdown group with received *irs* dsRNA (IRS). Bees in the GFP and IRS treatment groups were injected intra-abdominally between the fifth and sixth tergites using a Hamilton syringe fitted with a G30 needle (BD, Palo Alto, CA, USA). Injection volume was 3 μL. Injections took place over two days for each of two experimental colonies. Treated bees (n = 200 bees per treatment group, per strain, per colony) were tagged with numbered, plastic disks to allow for individual identification. Treated bees were introduced into one of two nucleus colonies with a background population of unselected, commercial stock bees, and allowed to recover from injection for two days. Then, each colony was transferred to a glass-walled observation hive. These hives were placed inside the lab and accessed the outdoors via a glass-topped runway.

#### 2.3. Knockdown Verification

We confirmed the efficacy of the *IRS* knockdown with quantitative real-time PCR. Abdominal fat bodies were dissected from seven-day old bees, flash frozen in liquid nitrogen, and stored at −80 °C until processing. We extracted RNA using TRIzol (GIBCO-BRL, San Diego, CA, USA)/chloroform extraction paired with RNeasy Blood and Tissue kit and an additional DNase treatment (Qiagen) as described previously [44]. RNA was quantified using two-step qRT-PCR, and analysed using the ΔΔCT method relative to expression of β-actin (GenBank: XM_623378) [50]. β-actin is stably expressed across several tissue types in adult honeybees and has been shown to be a reliable reference gene [51,52]. Samples were run in triplicate along with a negative control (a reaction lacking reverse transcriptase) to ensure reliability and the absence of DNA contamination. Knockdown efficiency was established by comparing relative expression of *irs* in the IRS and GFP treatment groups from both strains. This allowed us to determine the effect of reducing *irs* expression via targeted dsRNA versus the procedure of injecting dsRNA itself as described before [11,53,54].

#### 2.4. Age of First Foraging

In order to determine the age at which individual bees initiated foraging behaviour, we monitored the colony runways daily for two 30-min periods between 06:00 and 10:00 h. We recorded the individual tag IDs from all returning foragers as described before [41,49]. All observations were done in person. We used conservative criteria to determine age of first foraging for analysis [41,44]. Only bees that we observed more than once were included in the analysis, and age of first foraging was considered to be the first observation of an individual that was followed by a second observation within a seven-day period.

#### 2.5. Lifespan and Foraging Lifespan

We began colony censuses 10 days after injections were completed to exclude mortality due to injection trauma. The colonies were surveyed every other day after foraging activity had ceased to determine which bees were present in the hive. Each side of the two observation hives was scanned twice, and all tag IDs were recorded [41]. Only bees that were observed more than once were included in the analysis, and we considered a bee’s age of death to be the day after the last night she was observed [41]. Foraging lifespan was calculated as the number of days between the age of first foraging and age of death for an individual. Therefore, only those individuals that were observed to forage before death have a foraging lifespan.

#### 2.6. Statistics

Statistical analyses were performed using and R 3.5.1 (R Core Development Team 2017). Gene expression data were log-transformed to approximate normality before analysis [51,55]. Age of first foraging, foraging lifespan, and total lifespan data did not meet the assumptions of parametric or semi-parametric survival tests. Instead they were analysed using the Kaplan–Meier test [44,49]. To control for the effects of the replicated host colonies, we compared host observation colonies, using colony as factor and treatment as different strata for age of first foraging, foraging lifespan, and total lifespan. As these tests were all significant, were analyzed each replicate host colony separately. We then performed planned, pairwise comparisons between the GFP-injected control and *IRS* knockdown groups and between the *GFP* and non-injected NoI reference groups with log-rank tests. Results from the combined dataset are included in the supplemental materials. We used generalized linear mixed models (glmm) to assess the relationship of age of first foraging and foraging lifespan between treatment groups with host colony included as a random effect. Significance of fixed effects were evaluated using likelihood ratio tests. All data has been deposited in Dryad.

## 3. Results

### 3.1. Knockdown Verification

**Figure 1:**
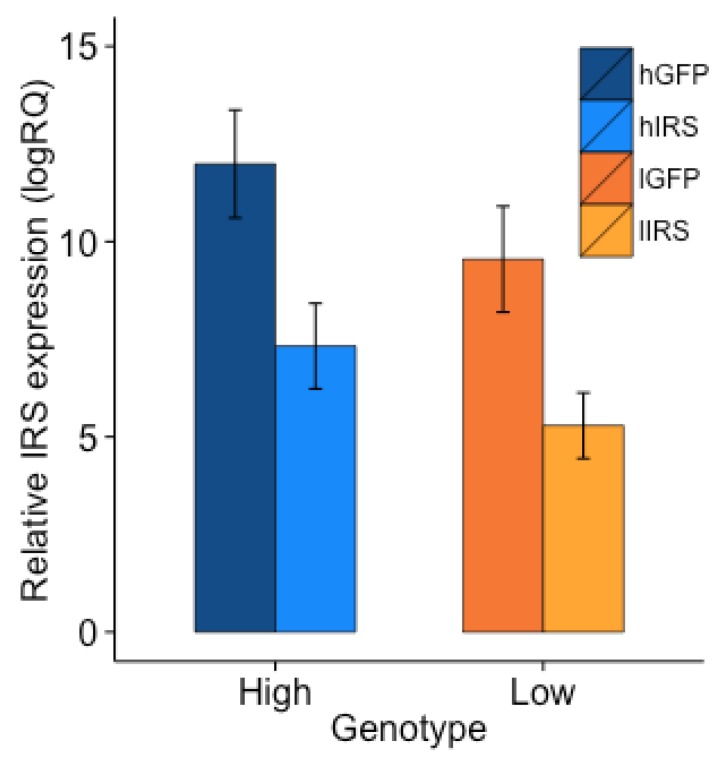
Expression of *irs* relative to *β-actin*. Bars are means ± s.e. *irs* expression was significantly reduced in both the high (Fisher’s LSD: high strain knockdown (hIRS, n = 18) vs high strain injected control (hGFP, n = 18), *p* = 0.0108) and the low (Fisher’s LSD: low strain knockdown (lIRS, n = 18) vs low strain injected control (lGFP, n = 18), *p* = 0.0359) strains following injection with dsRNA against the *irs* gene. High pollen hoarding workers had higher expression of *irs* than low strain workers. There was no interaction between treatment and genotype (F_2,63_ = 0.0037, *p* = 0.9514).

*irs* expression was influenced by treatment and genotype, but not by colony (factorial ANOVA: treatment, F_1,63_ = 11.4808, *p* = 0.0012 genotype, F_1,63_ = 4.9416, *p* = 0.0298; colony, F_1,63_ = 0.5440, *p* = 0.4635; Figure 1). High pollen-hoarding strain workers had significantly higher *irs* expression than low strain bees. *irs* knockdown was also significant when the strains were considered independently (Fisher’s LSD: high strain, *p* = 0.0108; low strain, *p* = 0.0359; Figure 1). There was no interaction-effect between treatment and genotype (F_2,63_ = 0.0037, *p* = 0.9514; Figure 1).

### 3.2. Age of First Foraging

**Figure 2:**
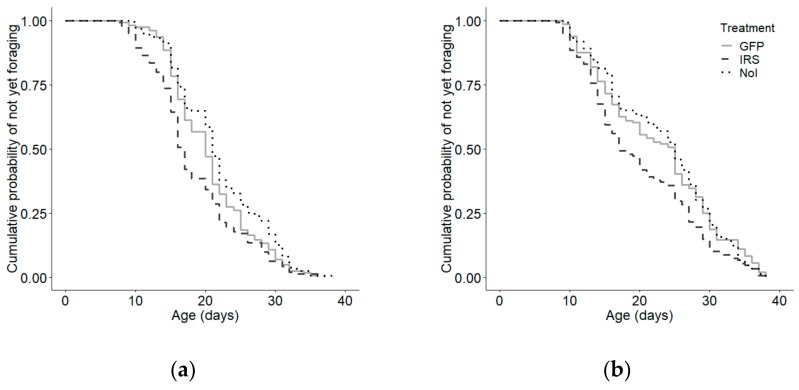
Effect of *irs* knockdown on age of foraging onset for all bees in (**a**) colony 1 and (**b**) colony 2. There was a significant overall effect of treatment on age of foraging onset in both colony 1 (Kaplan–Meier: χ^2^ = 15.3, *p* < 0.0001; n = 456) and (b) colony 2 (Kaplan–Meier: χ^2^ = 9.9, *p* = 0.007, n = 438). *irs* knockdown (IRS) induced an early onset of foraging behavior relative to injected GFP controls in both colonies (Cox–Mantel: Colony 1: *p* = 0.039, Colony 2: *p* = 0.011). There was no effect of handling or injection stress on age of first foraging (Cox–Mantel: Colony 1: *p* = 0.23, Colony 2: *p* = 0.72).

*irs* knockdown induced early foraging behaviour relative to GFP controls (Kaplan–Meier: χ^2^ = 33.089, *p* < 0.0001; n = IRS:287, GFP:302, NoI:305; Cox–Mantel: U = −34.759, *p* < 0.002; Figure S1). However, host observation colony had a significant effect on age of first foraging (Kaplan–Meier: χ^2^ = 25.2, *p* < 0.0001. n = 894), so we examined the response to treatment on age of first foraging within each observation colony separately. There was an overall effect of treatment in each colony (colony 1: Kaplan–Meier: χ^2^ = 15.3, *p* = 0.0005 (Figure 2a); colony 2: Kaplan–Meier: χ^2^ = 9.9, *p* = 0.007 (Figure 2b). As in the combined data set, the IRS knockdown group foraged significantly earlier than did the GFP-injected control group in both colonies (Cox–Mantel: colony 1: *p* = 0.039; colony 2: *p* = 0.011). There was no effect of handling and injection in either colony (Cox–Mantel: GFP vs NoI, colony 1: *p* = 0.055; colony 2: *p* = 0.984).

**Figure 3:**
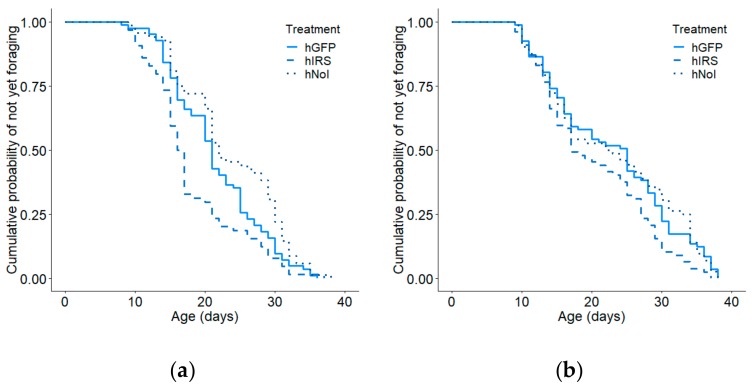
Effect of *irs* knockdown on age of first foraging in high pollen hoarding strain bees in (**a**) colony 1 and (**b**) colony 2. We observed an overall effect of treatment in the high strain for both colony 1 (Kaplan–Meier: χ^2^ = 16.8, *p* = 0.0002; n = 214) and (b) colony 2 (Kaplan–Meier: χ^2^ = 7.1, *p* = 0.028, n = 230). The *irs* knockdown group (hIRS) foraged significantly earlier than did the GFP-injected control group (hGFP) in both colonies (Cox–Mantel: Colony 1: *p* = 0.039, Colony 2: *p* = 0.011). No effects of handling and injection were observed (Cox–Mantel: GFP vs non-handled reference (hNoI), Colony 1: *p* = 0.055, Colony 2: *p* = 0.984).

**Figure 4:**
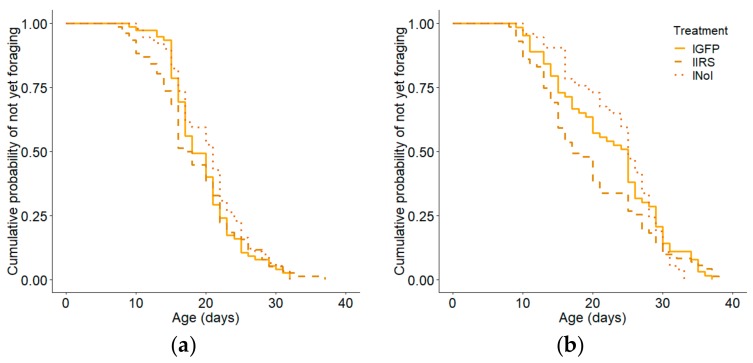
Effect of irs knockdown on age of first foraging in low pollen hoarding strain bees in (**a**) colony 1 and (**b**) colony 2. There was no effect of treatment in either colony 1 (Kaplan–Meier: χ^2^ = 2.0, *p* = 0.37; n = 242) or colony 2 (Kaplan–Meier: χ^2^ = 7.1, *p* = 0.028, n = 230).

When the high and low strains were considered separately by colony, the effect was overall in only significant the high strain (colony 1: Kaplan–Meier: χ^2^ = 16.8, *p* = 0.0002; n =214, Cox–Mantel: hIRS vs hGFP: *p* = 0.026, Figure 3a; colony 2: Kaplan–Meier: χ^2^ = 7.1, *p* = 0.028, n = 230, Cox–Mantel: hIRS vs hGFP: *p* = 0.025, Figure 3b). In the low strainthe effect was significant in colony 2, but not colony 1(colony 1: Kaplan–Meier: χ^2^ = 2.0, *p* = 0.37, n = 242 Figure 4a, colony 2: Kaplan–Meier: χ^2^ = 7.1, *p* = 0.028, n = 230, Figure 4b). The effects of handling stress on age of first foraging were significant only in colony 1 in the high pollen hoarding strain (Cox–Mantel: hGFP vs. hNoI: colony 1: *p* = 0.039, colony 2: *p* = 0.917; lGFP vs lNoI: colony 1: *p* = 0.23, colony 2: *p* = 0.72). hIRS bees-initiated foraging at a median age of 17 days while hGFP and hNoI had median ages of first foraging of 21 and 22 days respectively (Table S1). Median ages of first foraging were similar in the slow strain treatment groups at 17, 20, and 22 days for lIRS, lGFP and lNoI (Figure S2, Table S1).

### 3.3. Total Lifespan and Foraging Lifespan

**Figure 5:**
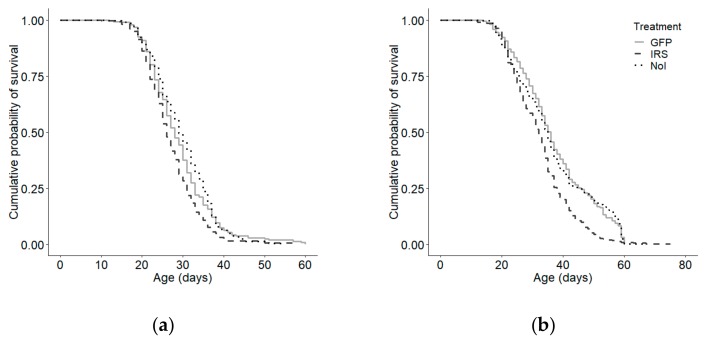
Effect of *irs* knockdown on total lifespan for all bees by host colony. (**a**) *irs* knockdowns (IRS) foraged earlier than injected controls (GFP: Kaplan–Meier: χ^2^ = 17.1, *p* = 0.0001 n = 857, Cox–Mantel: *p* = 0.0041). There was no effect of handling stress when the GFP group was compared with the non-handled reference group (NoI: Cox–Mantel: *p* = 0.37). (**b**) The same pattern was observed in colony 2 (Kaplan–Meier: χ^2^ = 34.4, *p* < 0.0001, n = 1114, Cox–Mantel: IRS vs GFP *p* = 0.001, GFP vs NoI *p* = 0.73).

Total lifespan was reduced by *irs* knockdown (Kaplan–Meier: χ^2^ = 23.178, *p* < 0.0001; n = IRS:618, GFP:665, NoI:688; Cox–Mantel: U = 88.349, *p* < 0.0001; Figure S3). There was also a significant effect of host colony on total lifespan (Kaplan–Meier: χ^2^ = 180, *p* < 0.000, n = 1971), so we again considered the observation colonies separately. As in the combined data set the *irs* knockdown group had significantly shorter lifespans than did GFP-injected controls in both colonies (colony 1: Kaplan–Meier: χ^2^ = 17.1, *p* = 0.0002, n = 857, Cox–Mantel: *p* = 0.0041, Figure 5a; colony 2: Kaplan–Meier: χ^2^ = 34.4, *p* < 0.0001, n = 1114, Cox–Mantel: *p* = 0.001, Figure 5b). There was no effect of handling or injection in either colony (Cox–Mantel: GFP vs NoI, colony 1: *p* = 0.37; colony 2: *p* = 0.73).

**Figure 6:**
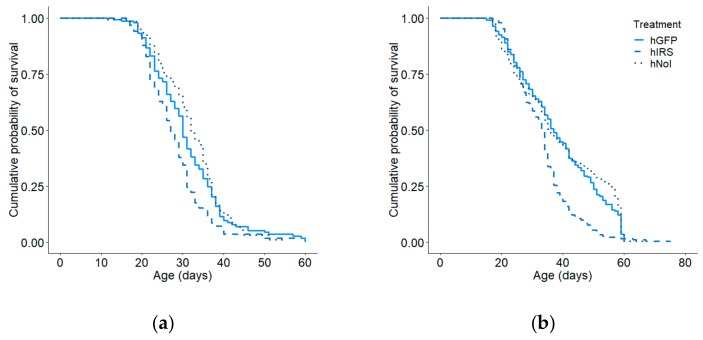
Effect of *irs* knockdown on total lifespan for the high pollen hoarding strain by host colony. In the high strain, *irs* knockdown (hIRS) resulted in decreased lifespan relative to the *GFP* control (hGFP) in both colonies (**a**) colony 1: Kaplan–Meier: χ^2^ = 14.9, *p* = 0.0006, n = 411, Cox–Mantel: *p* = 0.0052, (**b**) colony 2: Kaplan–Meier: χ^2^ = 28.3, *p* < 0.0001, n = 638, Cox–Mantel: *p* = 0.001. There was no effect of handling on total lifespan in the high strain (Cox–Mantel: hGFP vs. hNoI, colony 1: *p* = 0.482, colony 2: *p* = 0.52).

**Figure 7:**
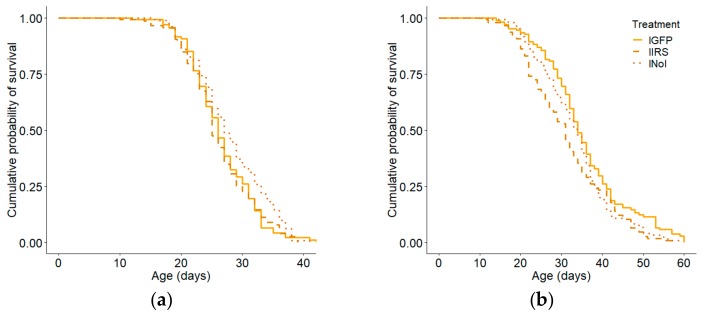
Effect of *irs* knockdown on total lifespan for the low pollen hoarding strain by host colony. In the low strain, the effect of *irs* knockdown (lIRS) relative to GFP-injected control (lGFP) on total lifespan was not significant in (**a**) colony 1 (Kaplan–Meier: χ^2^ = 5.9, *p* = 0.051, n = 446, Cox–Mantel: *p* = 0.054). (**b**) In colony 2 *irs* knockdown resulted in decreased total lifespan (Kaplan–Meier: χ^2^ = 10.6, *p* = 0.0051, n = 476, Cox–Mantel: *p* = 0.002).

When the high and low strains were considered separately, total lifespan was significantly reduced in the *irs* knockdowns relative to GFP-injected controls in the high strain in both colonies (Kaplan–Meier: χ^2^ = 14.9, *p* = 0.0006, n = 411, Cox–Mantel: *p* = 0.0052, Figure 6a, colony 2: Kaplan–Meier: χ^2^ = 28.3, *p* < 0.0001, n = 638, Cox–Mantel: *p* = 0.001, Figure 6b). In the low strain, *irs* knockdown reduced lifespan relative to GFP-injected control in colony 2 (Kaplan–Meier: χ^2^ = 10.6, *p* = 0.0051, n = 476, Cox–Mantel: *p* = 0.002, Figure 7b) but not in colony 1 (Kaplan–Meier: χ^2^ = 5.9, *p* = 0.051, n = 446, Cox–Mantel: *p* = 0.054, Figure 7a). There was no effect of handling for either strain in either colony (Cox–Mantel: hGFP vs. hNoI, colony 1: *p* = 0.482, colony 2: *p* = 0.52, Figure 6a,b, lGFP vs. lNoI, Colony 1: *p* = 0.054, colony 2: *p* = 0.054, Figure 7a,b). The median lifespan for hIRS bees was 28 days compared to 30 for hGFP and 27.5 for hNoI (Table S1). In the low strain, median lifespan was 24 days for the lIRS group and 27 days for the lGFP and lNoI groups (Figure S4, Table S1).

In contrast to both age of first foraging and total lifespan, foraging lifespan, the length of time between the first foraging flight and death, was not affected by *irs* knockdown (Kaplan–Meier: χ^2^ = 2.480, *p* = 0.289; n = IRS:285, GFP:301, NoI:305). Again, the results for the individual colonies were consistent with the overall results for foraging lifespan. We observed no significant effects of treatment on foraging lifespan in either colony in separate analyses (Kaplan–Meier: Colony 1: *p* = 0.84, Colony 2: *p* = 0.48). The same pattern was observed when we considered the strains separately (High strain, Colony 1: *p* = 0.82 (S2B), Colony 2: *p* = 0.83, Low strain, Colony 1: *p* = 0.92, Colony 2: *p* = 0.66.

### 3.4. Relationship between Age of First Foraging and Lifespan Components

Age of first foraging was significantly correlated with total lifespan (Pearson’s Correlation: overall: r = 0.616, *p* < 0.001, n = 891, colony 1: r = 0.684, *p* < 0.001, n = 456, colony 2: r = 0.58, *p* < 0.001, n = 435) as expected. Because age of first foraging has repeatedly been shown to be an important determining factor of total lifespan, we next examined the effect of treatment on total lifespan while controlling for age of first foraging. There was no effect of treatment on lifespan independent from age of foraging onset, but we did observe a significant treatment by age of first foraging effect on lifespan (glmm: age of first foraging: 0.911 ± 0.072 s.e., χ^2^ = 517.95, *p* < 0.0001; treatment (IRS v GFP): 0.853 ± 0.239 s.e., χ^2^ = 0.225 *p* = 0.894; age of first foraging x treatment: χ^2^ = 10.09, *p* = 0.0043). When the strains were considered independently, we observed the same pattern in the high strain (glmm: age of first foraging:0.90 ± 0.081 s.e., χ^2^ = 247.7, *p* < 0.0001; treatment (hIRS vs hGFP):8.576 ± 2.702 s.e., χ^2^ = 0.314, *p* = 0854; age of first foraging x treatment: χ^2^ = 12.81, *p* = 0.0016). In the low strain, we found no effects of treatment (glmm: age of first foraging: 0.787 ± 0.0786 s.e., χ^2^ = 303.58, *p* < 0.0001; treatment (lIRS vs lNoI): 1.18 ± 2.21 s.e. χ^2^ = 0.51, *p* = 0.775; age of first foraging x treatment: χ^2^ = 1.17, *p* = 0.559).

**Figure 8:**
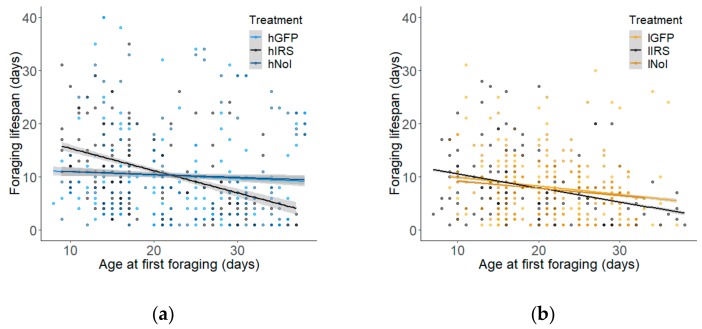
Relationship between pre- and post-foraging lifespan components predicted via the general linear model. (**a**) *irs* knockdown (hIRS) influences the relationship between the pre- and post-foraging components of lifespan in the high strain by reducing foraging lifespan more than the injected control (hGFP) group for each day that foraging onset was delayed (glmm: 0.39 ± 0.11 s.e., χ^2^ = 14,31, *p* = 0.0008, n = 444). (**b**) The interaction between age of first foraging and treatment was not significant in the low strain (glmm: 0.08 ± 0.101 s.e., χ^2^ = 1.12, *p* = 0.573, n = 447). Fitted lines are ± 95% confidence intervals.

Age of first foraging was also significantly correlated with foraging lifespan, albeit negatively (Pearson’s Correlation: r = −0.17, *p* < 0.0001, n = 891, colony 1: r = −0.288, *p* < 0.001, n = 456, colony 2: r = −0.165, *p* < 0.001, n = 435). We again tested the effect of knockdown on foraging lifespan while controlling for age of first foraging and found no effect of treatment independent of age of first foraging. We did observe a significant interaction effect (glmm: age of first foraging: 0.083 ± 0.695 s.e. χ^2^ = 40.20, *p* < 0.0001; treatment (IRS vs GFP): 8.202 ± 2.314 s.e., χ^2^ = 0.020, *p* = 0.894; age of first foraging x treatment: χ^2^ = 12.86, *p* = 0.0016). When the strains are considered independently, the interaction effect is significant in the high strain (glmm: age of first foraging: −0.093 ± 0.786, χ^2^ = 15.01, *p* = 0.0001; treatment (hIRS vs hGFP): 8.243 ± 2.614 χ^2^ = 0.27, *p* = 0.879; age of first foraging x treatment: χ^2^ = 15.78, *p* = 0.0004; Figure 8a), but not in the low strain (glmm: age of first foraging: −0.191 ± 0.077, χ^2^ = 26.70, *p* < 0.0001; treatment (lIRS vs lGFP): 1.21 ± 2.15 s.e., χ^2^ = 0.38, *p* = 0.989; age of first foraging x treatment: χ^2^ = 1.12, *p* = 0.573, Figure 8b).

Thus, *irs* knockdown changes the relationship between the pre-foraging and post-foraging initiation (foraging lifespan) portions of total lifespan in the high strain. For each additional day foraging onset is delayed, foraging lifespan decreases by 0.0.39 more days in the high strain *irs* knockdown group relative to the hGFP control group (glmm: 0.39 ± 0.11 s.e.). In the low strain *irs* knockdown group, foraging lifespan is reduced by only 0.08 days relative to the GFP control group (glmm: 0.08 ± 0.101 s.e.) with each day of delay in foraging onset.

## 4. Discussion

Unlike findings in many other organisms (reviewed in [13,56]), suppression of the IIS pathway does not extend lifespan in honeybee workers. On the contrary, *irs* knockdown results in decreased lifespan. Both across our study sample and within the high and low stains separately, *irs* knockdown workers had shorter lifespans than control bees. Much of this result can be explained by the effect of *irs* knockdown on behavioural maturation. Honeybee worker lifespan is negatively correlated with age at foraging onset, and the age at which a worker begins to forage is a strong determinant of total lifespan [24,27]. We found that *irs* knockdown induced early onset of foraging behaviour overall and within the high strain. In the low strain, the effect of *irs* knockdown on foraging onset was significant in only one colony, and while there was a consistent trend in the second colony the effect was not significant. In the overall dataset, the effect was significant through the 36th day of the 38-day foraging portion of the study period. Thus, low strain *irs* knockdowns were more likely to begin foraging at relatively early ages than were control bees, but this relationship begins to reverse at older ages. The behaviour and presence of nestmates is known to influence the timing of foraging onset in honey bees [57]. As such, it is possible that the differences between the ages of first foraging for our treatment groups can be exaggerated as a large cohort of *irs* knockdown bees foraging early, could act to further delay the foraging onset of the control groups.

We also observed limited effects due to what is likely handling and injection stress when we compared the non-injected reference group to the dsGFP-injected control group. As accelerated behavioural development and shorter lifespans are typical responses to a variety of stressors in honey bees [23,27,28,32], this result was expected. However, as the reference group was not available for the knockdown verification, we cannot rule out the contribution of off target effects of the dsGFP injection on *irs* expression. However, we believe this is unlikely as a study designed to document such effects across multiple data sets found no differences in *irs* expression due to dsGFP treatment [58].

A regulatory role for IIS in behavioural maturation and lifespan has been previously suggested by correlational data [15,17,59]. *AmIlp1* and *AmIlp2* have differential expression in nurses and foragers [15,22,23] and are correlated with known regulators of behavioural maturation, *vitellogenin* and juvenile hormone (JH) respectively [17]. In dipterans, and mosquitos in particular, the relationship between IIS, Vg, JH and lifespan is particularly well studied. Synthesis of JH is triggered by the IIS/TOR pathways and is, in turn, required for vitellogenesis [60,61]. Increased activity of this pathway reduces lifespan. In honeybees, these relationships are more complex. Vg interacts with the systemic JH in a mutually repressive feedback loop [62,63], with high titres of Vg associated with nursing behaviour and high titres of JH associated with foraging behaviour. Vg has also been demonstrated to impact lifespan both via its effects on behaviour [41,43,44] and through independent pathways associated with immune and antioxidant function [25,64]. The IIS pathway has been hypothesized to regulate JH signalling [15,21] and its interaction with Vg [49,65] in honeybees. These relationships are not clear-cut, and support for a direct regulatory role for IIS on JH, aging and lifespan, similar to that observed in dipterans, has remained elusive. Our results provide the first direct evidence that an IIS pathway gene influences behavioural maturation and lifespan in honeybees, and is consistent with previous work demonstrating that nutrient restriction and suppression of the nutrient sensing TOR pathway also result in precocious foraging behaviour [15,35,36,37].

However, accelerated behavioural ontogeny cannot fully explain the decreased lifespan of *irs* knockdown bees. While *irs* knockdown had no effect on total lifespan or foraging lifespan independent of age of first foraging, we did observe a significant interaction effect between these two outcomes. The total lifespan of a honeybee worker can be divided into pre- and post-foraging initiation components, which are separated by the age of foraging onset. The lengths of these life stages are negatively correlated [27,66]. Workers who begin foraging at earlier ages tend to have longer foraging lifespans than bees that begin foraging later [24,67]. The strength and slope of this correlation can be a measure of the effect of behavioural maturation on lifespan and the relative costs of pre- and post-foraging behaviours on total lifespan [27]. *irs* knockdown changed the relationship between the two behavioural states, both strengthening the correlation and the negative relationship between pre- and foraging lifespan. Thus, the *irs* knockdown induced shorter foraging lifespans than would have been expected if the overall decrease in total lifespan could be completely explained by an early age of foraging onset alone. Targeted future studies are needed to understand this novel effect of *irs* on lifespan.

The treatment by age of first foraging effects on total and foraging lifespan are significant only in the high strain, although the low strain exhibits similar non-significant trends. The genotype-dependent differences we observed are consistent with previous findings suggesting that the low pollen hoarding strain may be more behaviourally robust to single gene manipulations or, more narrowly, to perturbations of the IIS/JH/Vg signalling network, which appears to jointly regulate many of the traits differentially expressed by the high and low strains including foraging onset and food collection behaviours [11,44,65,68]. The low pollen hoarding strain is behaviourally insensitive to *Vg* knockdown, despite efficient reductions in *Vg* transcript. This effect is likely due to a decoupling of the relationship between Vg and JH in that strain [44]. This suggests that the more robust response to knockdown seen in the high strain bees may be more similar to that of unselected commercial stocks, as seen following *Vg* knockdown [44], but this remains to be tested. Strain-specific results were also identified in an earlier study that examined the effect of *irs* knockdown on food collection behaviour [11]. The high and low strain *irs* knockdowns increased their protein (pollen) relative to carbohydrate (nectar) collection, but they did it in different ways. The high strain increased pollen loading without decreasing nectar loading, while the low strain simply decreased nectar loading following *irs* knockdown [11]. It was hypothesized that this strain-specific response was mediated by *Vg* expression which is naturally lower in the low strain compared to the high strain, as Vg is associated with pollen foraging [41,44].

The strain-specific responses to *irs* knockdown observed in this study in the pacing of behavioural maturation and mortality may similarly be related to Vg and its role as a regulator of behavioural maturation and lifespan, They could also represent genotype-dependent differences in plasticity of the IIS/Vg/JH signalling network which favour increased responsiveness to individual physiology in the high strain, and phenotypic stability in the low strain. Future studies that include whole-transcriptomic responses to gene knockdown are needed to better understand how gene manipulations interact with individual genotype and colony phenotype to influence complex behaviour in a social animal.

## 5. Conclusions

In most organisms, dietary restriction and suppression of nutrient sensing pathways typically elicit a conserved extension of lifespan (reviewed in [13,56]). In contrast, we found that suppression of *irs* in free-flying honeybees results in a decrease in total lifespan, and that this decreased lifespan is determined largely by an early onset of foraging behaviour. Curiously, some studies on caged workers in the laboratory suggest that the conserved relationship between diet and lifespan may be intact when individual bees are deprived of a full social environment and behavioural repertoire [28,29,30,31]. However, there is disagreement between studies with several suggesting that this may be an artefact of artificial diets or experimental design [32,33,34]. Our results highlight the importance of considering the normal social environment of an individual when investigating the regulation of aging and lifespan in social animals.

## Supplementary Materials

The following are available online at https://www.mdpi.com/2075-4450/10/11/390/s1, Figure S1: Effect of IRS irs knockdown on age of foraging onset for all bees. There was a significant overall effect of treatment on age of foraging onset, Figure S2: Effect of *irs* knockdown on age of first foraging by strain. (a) We observed an overall effect of treatment in the high strain, Figure S3: Effect of *irs* knockdown on total lifespan for all bees. There was an overall effect of treatment on total lifespan for all bees, Figure S4: Effect of *irs* knockdown on total lifespan by strain. (A) In the high strain, *irs* knockdown resulted in decreased lifespan relative to the *GFP* control, Table S1: Descriptive statistics for age of first foraging and lifespan components data
