# Supplementary material for: Insulin Receptor Substrate Gene Knockdown Accelerates Behavioural Maturation and Shortens Lifespan in Honeybee Workers
Source: Insects. 2019 Nov 5;10(11):390. doi: 10.3390/insects10110390 (PMC6920892; doi:10.3390/insects10110390)
Supplement: Supplementary file 1 [file insects-10-00390-s001.pdf]

## Supplemental materials

## Survival trends of combined colonies

### *Age of first foraging*

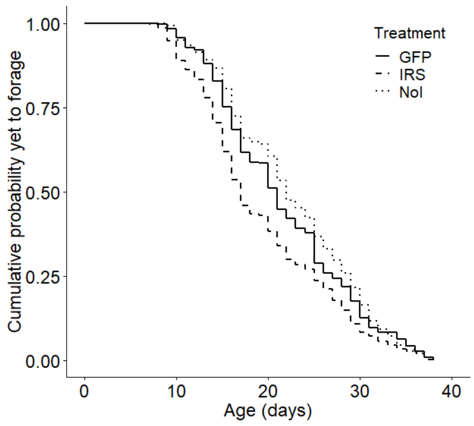


**Figure S1.** Effect of IRS irs knockdown on age of foraging onset for all bees. There was a significant overall effect of treatment on age of foraging onset (Kaplan-Meier: $\chi 2 = 33.089$, $p < 0\ .0001$; n =irs knockdown (IRS):287, GFP injected control (GFP):302, non-injected reference (NoI):305). IRS irs knockdown induced an early onset of foraging behavior relative to injected GFP controls (Cox-Mantel: U = -34.759, $p < 0.002$). There was no effect of handling or injection stress on age of first foraging (Cox-Mantel: U = 14.607, p = 0.206).

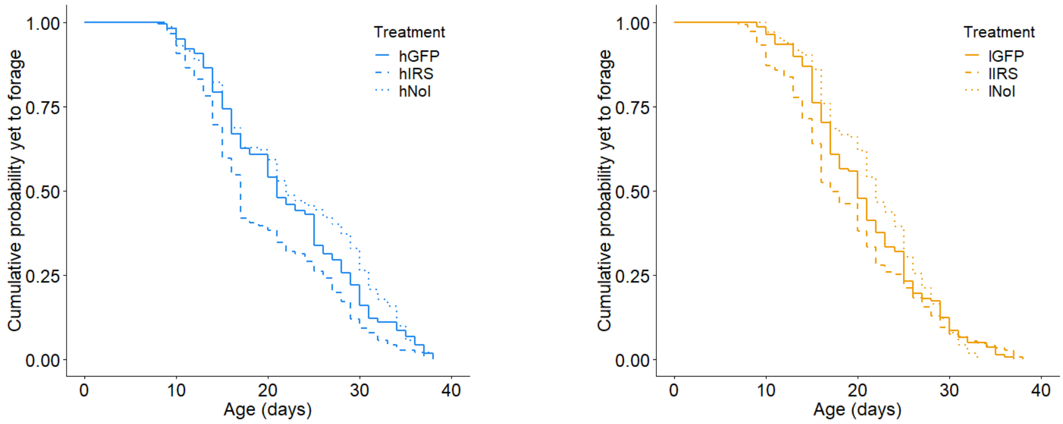


(**a**) (**b**)

**Figure S2.** Effect of *irs* knockdown on age of first foraging by strain. a) We observed an overall effect of treatment in the high strain (Kaplan-Meier: $\chi^2 = 16.166$, $p < 0\ .002$; n = high strain *irs* knockdown (hIRS):140, high strain GFP injected control (hGFP):163, high strain non-injected reference (hNoI):140), and *irs* knockdown induced early foraging onset in high strain workers (Cox-Mantel: U = 22.845, $p < 0.004$). b) While there was an overall effect of treatment in the low strain Kaplan-Meier: $\chi^2 = 18.044$, p = 0 .0001; n = low strain *irs* knockdown (lIRS):147, low strain GFP injected control (lGFP):139, low strain non-injected reference (lNoI):165), *irs* knockdown had no significant effect on the age of foraging onset in this strain (Cox-Mantel: U = -4.468, p = 0.234).

*Total lifespan*

Total lifespan was reduced by *irs* knockdown (Kaplan-Meier: $\chi^2 = 23.178$, $p < 0.0001$; n = IRS:618, GFP:665, NoI:688; Cox-Mantel: U = 88.349, $p < 0.0001$; Figure S3). Handling stress and injection of dsRNA in general had no effect on total lifespan (Cox-Mantel: U = 4.567, p = 0.767; Figure S3). Further, *irs* knockdown reduced lifespan in both the high strain (Kaplan-Meier: $\chi^2 = 14.21$, p = 0 .005; n = hIRS:320, hGFP:351, hNoI:378; Cox-Mantel: U = -48.724, $p < 0.0001$; Figure S4a) and the low strain (Kaplan-Meier: $\chi^2 = 12.927$, p = 0.0016; n = lIRS:298, lGFP:314, lNoI:310; Cox-Mantel: U = -33.646, $p < 0.0008$; Figure S4b). There was no effect of handling and injection of lifespan in either strain (High strain: Cox-Mantel: U = 10.485, p = 0.346; Low strain: U = -18.915, p = 0.0711; Figure S4a,b).

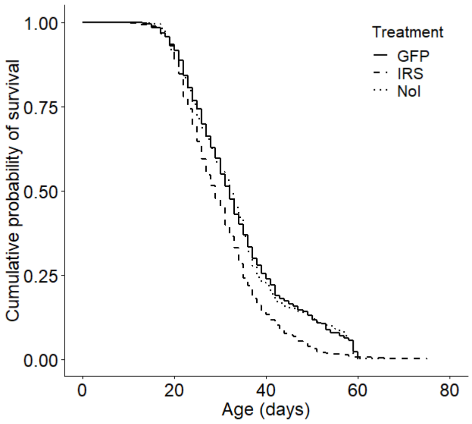


**Figure S3.** Effect of *irs* knockdown on total lifespan for all bees. There was an overall effect of treatment on total lifespan for all bees (Kaplan-Meier: $\chi^2 = 23.178$, p <0 .0001; n = *irs* knockdown (IRS):618, GFP injected controls (GFP):665, non-injected reference (NoI):688). *irs* knockdowns had significantly shorter lives than *GFP* (Cox-Mantel: U = 88.349, $p < 0.0001$). There was no significant effect of handling or injection on total lifespan (Cox-Mantel: U = 4.567, p = 0.767).

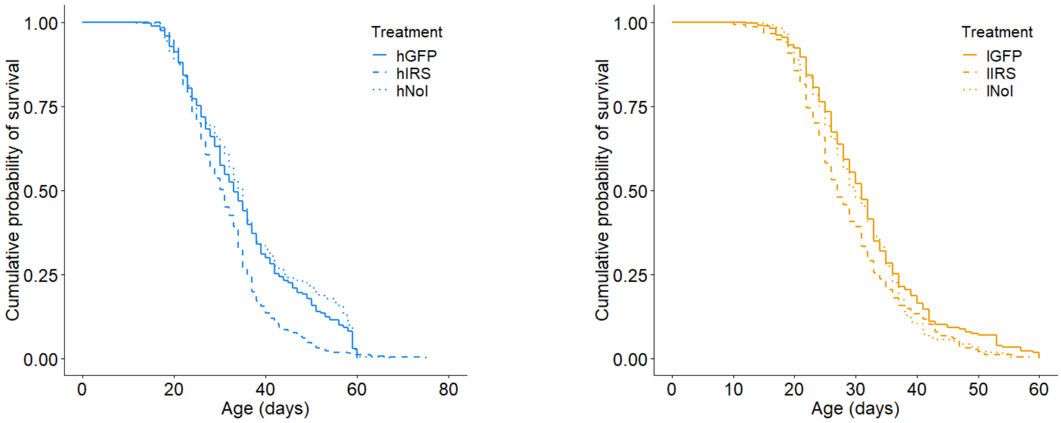


(**a**) (**b**)

**Figure S4.** Effect of *irs* knockdown on total lifespan by strain. A) In the high strain, *irs* knockdown resulted in decreased lifespan relative to the *GFP* control (Kaplan-Meier: $\chi^2 = 14.21$, p = 0 .005; n = high strain *irs* knockdown (hIRS):320, high strain GFP injected control (hGFP):351, high strain non-injected reference (hNoI):378; Cox-Mantel: U = -48.724, $p < 0.0001$). There was no effect of handling on total lifespan in the high strain (Cox-Mantel: U = 10.485, p = 0.346). B) In the low strain, *irs* knockdown also induced decreased lifespan relative to *GFP* controls (Kaplan-Meier: $\chi^2 = 12.927$, p = 0.0016; n = low strain *irs* knockdown (lIRS):298, low strain GFP injected control (lGFP):314, low strain non-injected reference (lNoI):310; Cox-Mantel: U = -33.646, $p < 0.0008$). We again observed no effect of handling on lifespan in this strain (Cox-Mantel: U = -18.915, p = 0.0711).

In contrast to both age of first foraging and total lifespan, foraging lifespan, the length of time between the first foraging flight and death, was not affected by irs knockdown (Kaplan-Meier: $\chi 2$ = 2.480, p = 0.289; n = IRS:285, GFP:301, NoI:305). irs knockdown did not affect foraging lifespan in either the high strain (Kaplan-Meier: $\chi 2 = \chi 2$ = 3.596, p = 0.166; n = hIRS:140, hGFP:163, hNoI:140) or the low strain (Kaplan-Meier: $\chi 2$ = 0.1333, p = 0.9355; n = lIRS:144, lGFP:138, lNoI:165).

**Table 1.** Descriptive statistics for age of first foraging and lifespan components data.

| | | | **Age of First Foraging** | | | |
|---|---|---|---|---|---|---|
| **Strain** | **Treatment** | **N** | **Mean (d)** | **Standard Error (d)** | **Median (d)** | **Interquartile Range (d)** |
| High pollen hoarding | GFP | 163 | 22.15 | 0.62 | 21 | 14 |
| | IRS | 141 | 19.40 | 0.63 | 17 | 12 |
| | NoI | 140 | 23.24 | 0.72 | 22 | 16 |
| Low pollen hoarding | GFP | 138 | 20.92 | 0.56 | 20 | 9 |
| | IRS | 147 | 19.17 | 0.60 | 17 | 10.5 |
| | NoI | 165 | 21.96 | 0.47 | 22 | 10 |
| | | | **Lifespan** | | | |
| **Strain** | **Treatment** | **N** | **Mean (d)** | **Standard Error (d)** | **Median (d)** | **Interquartile Range (d)** |
| High pollen hoarding | GFP | 351 | 32.25 | 0.69 | 30 | 17 |
| | IRS | 320 | 29.42 | 0.55 | 28 | 13 |
| | NoI | 378 | 30.91 | 0.72 | 27.5 | 20 |
| Low pollen hoarding | GFP | 314 | 28.65 | 0.56 | 27 | 13 |
| | IRS | 298 | 25.81 | 0.52 | 24 | 11 |
| | NoI | 310 | 28.46 | 0.48 | 27 | 12.75 |
| | | | **Foraging Lifespan** | | | |
| **Strain** | **Treatment** | **N** | **Mean (d)** | **Standard Error (d)** | **Median (d)** | **Interquartile Range (d)** |
| High pollen hoarding | GFP | 163 | 10.16 | 0.67 | 8 | 10 |
| | IRS | 141 | 11.40 | 0.72 | 10 | 11 |
| | NoI | 140 | 10.24 | 0.73 | 7 | 11 |
| Low pollen hoarding | GFP | 138 | 8.12 | 0.58 | 7 | 8.75 |
| | IRS | 144 | 8.14 | 0.52 | 7 | 7 |
| | NoI | 165 | 7.56 | 0.39 | 7 | 8 |
